# Vitamin D and Chronic Diseases

**DOI:** 10.14336/AD.2016.1021

**Published:** 2017-05-02

**Authors:** Hanmin Wang, Weiwen Chen, Dongqing Li, Xiaoe Yin, Xiaode Zhang, Nancy Olsen, Song Guo Zheng

**Affiliations:** ^1^Division of Endocrinology, Quqing First Hospital at Kunming Medical University, Qujing, Yunan 655400, China; ^2^Expert Workstation, Quqing First Hospital at Kunming Medical University, Qujing, Yunan 655400, China; ^3^Division of Rheumatology, Milton S Hershey Medical Center at Penn State University, Hershey, PA17033, USA

**Keywords:** Vitamin D, chronic diseases, early prevention, deficiency

## Abstract

Vitamin D is one of the essential nutrients to sustain the human health. As a member of the steroid hormone family, it has a classic role in regulating metabolism of calcium and a non-classic role in affecting cell proliferation and differentiation. Epidemiological studies have shown that 25OHD deficiency is closely associated with common chronic diseases such as bone metabolic disorders, tumors, cardiovascular diseases, and diabetes. 25OHD deficiency is also a risk factor for neuropsychiatric disorders and autoimmune diseases. 25OHD deficiency is highly prevalent in the world. It is therefore necessary to know the adverse health effects of 25OHD deficiency, and to design interventions and early treatments for those who are likely to have low levels of 25OHD.

Many studies have demonstrated that Vitamin D has multiple effects on biological processes regulating calcium and phosphorus metabolism as well as effects on cell proliferation, differentiation, apoptosis, immune regulation, genome stability, and neurogenesis. Recent studies have also found that Vitamin D is closely associated with cardiovascular diseases, diabetes, cancers, autoimmune diseases, infectious diseases, and others [1, 2]. Many experts now believe that Vitamin D (especially with biological activities of 1, 25(OH)2D3) should be considered a hormone rather than one of the conventional nutritional vitamins.

## Vitamin D overview

Vitamin D in the human body is mainly derived from skin after ultraviolet light exposure and from dietary sources. Vitamin D is derived from 7-dehydrocholesterol, which is converted in the skin by ultraviolet light band B (UVB) to Vitamin D3 (cholecalciferol), an inactive precursor. More than 90% of systemic Vitamin D originates from the skin and around 10% from food intake [1].

There are two main forms of Vitamin D, Vitamin D3 (cholecalciferol) and Vitamin D2 (ergocalciferol). Following the absorption from the intestines or/and the synthesis by skin, Vitamin D is transferred to the liver where it is metabolically converted into 25(OH)D in the liver, 25 (OH)D has a further metabolic conversion in the kidney. The active metabolites 1, 25 (OH) 2D3 and 24R (OH) D can be converted by 25 (OH) 1α-hydroxy-D (CYP27B1) or 24R hydroxylase (CYP24A1 or CYP24) in the kidney, respectively.

Vitamin D, an essential nutrient to sustain health, is a member of the steroid nuclear hormone superfamily, and was first discovered to be able to prevent rickets in children [3]. Further research has found that Vitamin D has broader physiological functions. Currently the biological effects of Vitamin D are divided into two categories: First, in calcium and phosphorus metabolism, considered the classical activity; and second, the non-classical or alternative pathway that mainly affects immune function, inflammation, anti-oxidation, anti-fibrosis and others [4-7], as wells as inhibitory effects on the many kinds of malignancies [7-9].

1, 25 (OH) 2 D3 is an autocrine hormone. The 1α-hydroxy enzyme is also widely expressed in non-kidney cells, such as osteoblasts, monocytes, macrophages, neuron cells, pancreatic cells, breast cancer, and colon cancer cells. 25 (OH) D in these cells can be directly related to the active form of metabolic enzymes and is not affected by the classical hormonal regulation of calcium and phosphorus metabolism [10]. As 25 (OH) D is much more stable than 1,25(OH)2D3 in the body, 25 (OH)D has been considered as an important indicator of Vitamin D levels in humans [11].

Vitamin D has multiple functions and target organs. Vitamin D and the nuclear vitamin D receptor (VDR) after binding can influence the expression of many genes. VDR is widely expressed in the kidney, immune cells, bone cells and other cells [12]. Studies have shown that the levels and activities of Vitamin D are closely related to the occurrence and development of many chronic conditions, such as malignancies, autoimmune diseases, metabolic disorders, and infectious disease s[13].

Once formed, Vitamin D3 is ejected out of the keratinocyte plasma membrane and is drawn into the dermal capillary bed by Vitamin D Binding Protein (DBP). Vitamin D ingested is then incorporated into chylomicrons, which are released into the lymphatic system [2, 14], and enter the venous blood, where they bind to DBP and lipoproteins and are transported to the liver [14, 15]. The 25(OH)D bound to DBP is filtered by the kidneys and is reabsorbed in the proximal renal tubules by megalin cubilin receptors [14, 16].

25 (OH) D in circulating is mostly in 25-(OH) D-DBP complex form [17]. In a study of DBP gene knockout mice, DBP was shown to protect 25-(OH) D and 1, 25 (OH) 2 D 3 from degradation by kidney [18], to adjust the systemic levels of Vitamin D [19]. Additionally, susceptibility to a variety of malignant tumors including breast cancer, prostate cancer, colorectal cancer, is also related to VDBP reduction [20]. Other studies have found that 25OHD not only works through the VDR, but also through combining with Vitamin D dependent calcium binding protein [21]. Some of these studies have found that in islet beta cells, 25OHD can adjust the Vitamin D receptor and Vitamin D dependent calcium binding protein, thereby promoting synthesis and secretion of insulin [22, 23].

## The criteria for Vitamin D levels

Although there are different methods and criteria on defining Vitamin D levels, the criteria Holic proposed has been widely accepted. In this proposal, it is suggested as Vitamin D deficiency if the level of 25 (OH) D in circulating blood in human is less than or equal to 20 ng/ml (50 nmol/L), insufficiency if between 21 to 29 ng/ml and sufficiency if greater than or equal to 30 ng/ml [24].

Based on this criterion, adult 25OHD deficiency was found in both developing and developed countries. For example, about 40% population suffer from 25OHD deficiency in China in winter season, and the incidence of 25OHD deficiency is higher among women than in men, and higher in elderly group than young one [25]. In general, Chinese elderly population has a low level of Vitamin D with 25 (OH) D averaging of 14-16 ng/ml and Vitamin D deficiencies accounted for 83%-93%, of which about 30% have a serious shortage. Vitamin D level continues to decrease along increasing age [25]. Centers of Diseases Control and Prevention (CDC) reported that 32% of children and adults have a circulating concentration of 25OHD less than 20 ng/mL. Reports from Mexico, South America, Europe, Asia, India and Africa suggest that more than 50% of the world population is at risk for 25OHD deficiency [14, 15, 26]. High rates of biochemical 25OHD deficiency or insufficiency among healthy individuals have been reported in large-scale studies from all parts of the world: The United States [27], Canada [28], South America [29], Europe [30], Australia [31], the Middle East [32], South Asia [33], and Africa [34].

25OHD level is also generally low in infants, children and women in menopause. The proportions of deficiency and insufficiency among these populations vary between 30% to 80%, and even teenagers in the sunny plateau area. Recent study observed that population in sunny plate area still have an incidence of 25OHD deficiency between 23%-44% [35]. There is a low 25OHD level in the populations from developing countries even in sunny climates [36], suggesting that the 25OHD deficiency is not only due to the lack of sunshine.

## Relationship between 25OHD deficiency and a variety of chronic diseases

In recent years, accumulating epidemiological and laboratory evidence has documented that 25OHD deficiency is correlated to the onset and progression of many chronic diseases.

### Vitamin D and cancer

Vitamin D binding protein (VDBP) as a carrier of Vitamin D can be combined with Vitamin D and its metabolites to play a crucial role in transport to the cell. Deficiency in VDBP also affects the function of Vitamin D. For example, studies have demonstrated that low level of VDBP is related to a variety of malignant tumors, including breast, prostate and colorectal [20, 37-41]. Both prospective and retrospective epidemiologic studies indicate that levels of 25OHD below 20 ng/ml are associated with a 30 to 50% increased risk of incident colon, prostate, and breast cancer, along with higher mortality from these cancers [42-46]. The report that postmenopausal women who increased their vitamin D intake by 1100 IU of vitamin D 3 reduced their relative risk of cancer by 60 to 77% is a compelling reason to be vitamin D sufficient [47]. Similarly, a recent study observed that 25 (OH) D progressively decline during the course of the development of liver fibrosis, cirrhosis to liver cancer [37].

### Vitamin D and cardiovascular diseases

The pathogenesis of chronic cardiovascular disease is affected by a variety of risk factors. Clinical studies found that in addition to high cholesterol, smoking, obesity, high blood pressure and diabetes, low serum levels of 25OHD are also closely related to the occurrence of cardiovascular diseases [48]. In addition, hypertension incidence may be related to low levels 25OHD [49]. The role of vitamin D in the cardiovascular system is noticeable since the presence of its receptors not only the heart but also in the entire cardiovascular system [50]. The 1, 25(OH)2D3 active form of Vitamin D combines with VDR and then regulates the expression of many genes [51, 52].

Vitamin D metabolites act on multiple domains of cardiovascular function including those related to inflammation, thrombosis and the renin angiotensin aldosterone system (RAAS) pathways [53]. Vitamin D has well known effects in endothelial cells, where it stimulates nitric oxide (NO) production [54], protects against oxidative stress [58] and prevent endothelial apoptosis [55, 56] by diverse genomic and non-genomic pathways. As endothelial function has emerged as a key factor for initiation and progression of atherosclerotic process, vitamin D may contribute for reversing atherosclerosis burden [57]. A broad series of in vitro studies have also suggested that vitamin D and its analogs consistently suppress pro-inflammatory cytokines and increases anti-inflammatory cytokines, which mechanisms involved seem to be related to the inhibition of NF-kB and p38 pathways by VDRs [58].

Vitamin D also seems to present an anticoagulant activity [59], through the regulation of the expression of pro-coagulant and antifibrinolytic factors. Low levels of Vitamin D lead to the activation and releasing of pro-inflammatory cytokines which then increases the risk of CVD by mediating endothelial dysfunction and arterial wall stiffness [60]. Large epidemiological studies have shown that 25OHD deficiency is a signal of cardiovascular disease risk [61]. Mechanisms likely include the elimination of Vitamin D receptor in macrophage, which then triggers insulin resistance and mononuclear phagocytic cells of cholesterol, thereby accelerating atherosclerosis in mice [62].

### Vitamin D and diabetes

25OHD deficiency has been shown to be related to the development of diabetes [63]. Recent studies have shown that 25 (OH) D levels are negatively correlated to prevalence of diabetes mellitus type 2(T2DM), islet beta cell function, insulin resistance, body fat, and BMI levels [64, 65]. Conversely, 25(OH)D levels were positively correlated with insulin sensitivity. 25OHD deficient individuals have a higher insulin resistance and type 2 diabetes risk [66].

1, 25 (OH) 2 D3 can be combined with Vitamin D3 receptor on the islet β cells, increasing insulin sensitivity, inhibiting inflammatory factors, alleviating chronic inflammation process of the pancreas to improve the function of islet β cells [67]; Additionally, it also inhibits the action of the renin-angiotensin system, which promotes insulin secretion [68]. Vitamin D supplementation can improve islet β cell function and glucose tolerance [69].

Studies have found that Vitamin D dependent calcium binding protein is present not only in islet β cells, also in PP cells and D cells, and is most abundant in islet α cells [70]. Its principal action is to regulate the concentration of intracellular calcium by Vitamin D dependent calcium binding protein, thus affecting the endocrine and metabolic processes in the various cells of the pancreas. Other studies have found that glucagon hormone release was significantly higher in islets isolated from 25OHD deficient mice than in those isolated from mice with normal levels of Vitamin D, following in vitro stimulation with 10 mmol/L arginine or hypoglycemia (blood glucose control is less than 1.7 mmol/L). Moreover, supplementation with 1,25 (OH) 2D3 can make glucagon restored to normal levels [71]. Thus, 25OHD deficiency not only suppresses insulin secretion through weakening the islet β cell function, but also elevates blood sugar through enhancing islet α cell function.

### Vitamin D and immune system diseases

Vitamin D can play a role in regulating immune function, inhibiting inflammatory reactions and autoimmune diseases [72]. 1, 25(OH) 2D3 combined with Vitamin D receptor plays an important role in immune cell biology [73].

Many immune cells in the human body such as monocytes, macrophages, dendritic cells, T cells, and B cells express VDR. Vitamin D3 in combination with VDR in T cells can inhibit the activity of Th1 cells, thereby reducing the CD4 + T cells to release IL-2, interferon γ and tumor necrosis factor (TNF) α?β,and to delay the progress of chronic inflammatory autoimmune diseases [74]. In addition, it can promote the differentiation of mononuclear cells into macrophages, and strengthen their chemokine production and ability to control infection [75]. It also inhibits the major histocompatibility complex II (MHC II) expression on antigen-presenting cells to prevent the activation of immune system [76]. Vitamin D also induces B cell proliferation and the secretion of immunoglobulins E and M, leading to the formation of memory B cells and B cell apoptosis promotion [77]. These results have demonstrated that 1, 25-(OH)2D3 has an important role in the anti-inflammatory response and immune regulation.

### Vitamin D and neuropsychiatric disorders

Vitamin D is closely related to the metabolism of CYP27B1. CYP27B1 was known as CYP27B1. CYP27B1 is expressed in neurons and glial cells in both of fetus and adult, especially in the substantia nigra supra optic and hypothalamus paraventricular tissues. Additionally, VDR is highly expressed in the hypothalamus, pons, basal ganglia, hippocampus, as well as in developing brain tissues, suggesting that Vitamin D may be involved in the development and function of the brain [78].

It is likely that Vitamin D participates in the process of neurotransmitter synthesis, inflammation, and calcium balance. Other studies have documented that Vitamin D can protect nerve cells by its antioxidant effect [79]. A cohort study including 56366 American women between ages 50 to 79 years has shown that high levels of Vitamin D intake can significantly reduce the risk of depression, providing additional evidence to suggest that 25OHD deficiency affects the neuropsychiatric disorders [80].

There are a variety of similar studies relating 25OHD deficiency with increased risk for depression [81, 82], Alzheimer disease [83], epilepsy [84] and neurocognitive decline [85, 86]. Evidence suggests that 1,25(OH)2D3 could increase calcium binding protein expression [87], although this could not be shown in all studies [88]. 1,25(OH)2D3 could also act by increasing serotonin levels in the brain [89]. Furthermore 1,25(OH)2D3 has also been demonstrated to stimulate amyloid-β phagocytosis and clearance by macrophages in Alzheimer patients [90]. This may help explain the association between neurocognitive decline, dementia, depression, and Alzheimer diseases and a high prevalence of 25OHD deficiency [81, 82, 86, 90, 91].

### Vitamin D and other diseases

The outcome of 25OHD deficiency in terms of osteoporosis, osteomalacia and increased fracture risk is well known [92, 93]. Furthermore, vitamin D may be relevant in the physiopathology of chronic liver diseases because of its effect on the immune system and its anti-fibrotic effect [94-96]. A high percentage of chronic hepatitis C virus infection patients (46% to 92%) have low 25OHD levels [97-99], and more than 25% those were suffering from severe 25OHD deficiency [97-99]. In a recent clinical study of adults with non-alcoholic fatty liver disease (NAFLD), Targher et al [100] showed that the 25OHD levels had an effect on the development of hepatic steatosis and in the severity of the histological lesion. A possible role of vitamin D has also implicated in several other diseases, including multiple sclerosis [101], psoriasis [102], OA [103], and chronic kidney disease [104]. Taken together, 25OHD levels affect the multiple diseases.

## Conclusion

25OHD deficiency predominately affects the occurrence and development of many chronic diseases. With an aging population, the numbers of patients with osteoporosis, cardiovascular diseases, cancers, diabetes and other chronic diseases dramatically increase. Similarly, the incidence of neurological and psychiatric diseases is also increasing year by year. These outcomes result in the loss of life of the population, reduced quality of life and significant social and economic burdens. Since these chronic diseases lack specific treatment or the treatment effects are not curative, strategies for the control of chronic diseases should focus on the prevention. Vitamin D deficiency is associated with chronic disease. Hence, it is challenging to clarify whether vitamin D deficiency is the cause or only the consequence of various chronic diseases. We need to continue to study Vitamin D.
